# Increasing collaboration between health professionals: Clues and challenges

**Published:** 2015-06-30

**Authors:** Nelson Alberto Aguirre-Duarte

**Affiliations:** Medical Doctor and Surgeon. Master in Management and Business Administration. PhD in Health Sciences. The University of Auckland, New Zealand

**Keywords:** Integrated health care systems, Health networks, communication, collaboration, Coordination

## Abstract

**Background:**

Scholars have recently started to pay more attention in the potential of the inter-professional relationship between general practitioners and specialists to improve outcomes, through consideration given to the effect on prescribing practices. However, more empirical research is needed.

**Objective:**

To explore inter-professional network factors that may explain effects on General Practitioners prescription behaviours.

**Methods:**

A qualitative study was conducted in an integrated diabetes care program. Data was collected through semi-structured interviews from 16 health practices and a hospital diabetes clinic, using a convenience sample of general practitioners, practices nurses, diabetes nurse specialists and endocrinologists. A conceptual mapping was performed to identify factors underlying networks and effects on patient outcomes.

**Results:**

Four themes with their concepts emerged from the conceptual map. These demonstrated the need for building effective channels of communication to share experience and knowledge timely in diabetes care. Communication, collaboration and coordination are critical factors to influence prescription behaviours within primary and secondary care.

**Conclusions:**

conceptual mapping allowed understanding factors that might explain how links between health professionals can improve patient outcomes at the primary and secondary care interface.

## Abstract (es)

**Antecedentes:**

Recientemente, académicos están dedicando más atención al potencial de las relaciones inter-profesionales entre médicos generales y especialistas, para mejorar los resultados en los pacientes a través de mejores prácticas de formulación. Sin embargo, es necesaria más investigación empírica.

**Objetivo:**

Explorar los mecanismos asociados en la colaboración entre profesionales de salud en la interface entre el primero y segundo nivel de salud, y sus efectos los comportamientos de prescripción de los médicos.

**Métodos:**

Fue conducido un estudio cualitativo en el contexto de un programa integrado de atención de pacientes diabéticos. Fueron utilizadas entrevistas semi-estructuradas para la recolección de datos, en 16 centros de atención en salud y un programa de diabetes en el hospital de referencia, usando muestreo por conveniencia de médicos generales, enfermeras generales, enfermeras especialistas en diabetes y endocrinólogos.

**Results:**

Cuatro temas con sus respectivos conceptos emergieron del mapa conceptual, identificando de manera general factores asociados a la red de profesionales y sus efectos en los pacientes. Lo que demuestra la necesidad de crear efectivos canales de comunicación para compartir experiencias y conocimientos en el cuidado de los pacientes diabéticos de una forma oportuna. Comunicación, colaboración y coordinación son factores críticos que influencian los comportamiento de prescripción de los médicos generales en la interface entre el nivel de atención primario y secundario

**Conclusiones:**

El mapeo conceptual permitió un mejor entendimiento en como las interacciones entre profesionales de salud entre el primero y segundo nivel de salud puede mejorar la condición clínica de los pacientes.

## Introduction

As a result of the increased number of patients with chronic diseases such as diabetes, there is a pressure on the health systems worldwide in order to find cost-effective and patient-centered solutions 1. Strategies based on integrated care between primary and secondary care have been presented as a solutions in the developed countries 2. It is evidence based and well-accepted fact that an initial contact with general practitioners in primary care (before seeking for specialized treatment) is associated with an appropriate, more effective and less costly delivery of healthcare 3.

That aspect is a priority in countries such as New Zealand and Australia between others, and as a result there is an emphasis to invest resources in order to get better integration between primary and secondary care 4. That decision is based on the assumption that cooperation and better communication between health professionals increases the quality of patient care 5-9. Though it looks obvious that strong relationship between health professionals may be associated with better patient outcomes - it is necessary to provide evidence which must be based on empirical research. Current study was performed to fill this gap by investigating inter-professional relationships between general practitioners, nurses and specialists; from the health professionals' perspective to see how it affects patient outcomes and to explore the mechanisms which may explain this phenomenon.

### Background

The effects of social networks on outcomes - how interactions between people could modify the efficiency of the system, has been an area of research interest since last decade 10,11. The evidence to this supposition is based on results of empirical research in business and industrial settings 12-14. However, there is paucity of such evidence in the healthcare context, the reason why recent systematic reviews have pointed out the need to identify this relationship in this particular setting 15-17. Based on the assumption that a better communication between health professionals may positively affect the prescription behaviours and of course benefits for the clinical conditions of the patients.

A recent study at the integrated diabetes care context has clarified that there is a positive association between strong relationship among general practitioners and specialist and improved prescription behaviours 18,19. That study presents empirical evidence about the direct influence of the specialist on general practitioners' prescription behaviours. Additionally, it is explained how general practitioners strongly connected with specialists in secondary care had better access on timely manner to accurate information required to manage patients with diabetes leading to better prescription behaviours and improved patient outcomes.

The study also suggested that on the patient outcomes equation it is also necessary to take into account the communication and collaboration within multidisciplinary teams 18,20. The need to design more effective channels of communication between health professionals in order to integrate primary and secondary care interface is the practical implication. It is well known that one of the immediate consequences of the integrated care is to increase communication and coordination within levels of care; however it is also clear that to establish and maintain interactions through inter-professional networks is a challenge 21,22.

Primary and secondary care interface is a clear example where integration strategies need to be implemented, because more holistic care is provided, avoiding the effects of the fragmentation, duplication and adverse events. Nevertheless, human interactions are complex 4,23. Therefore, it is crucial to understand how health professionals faced the challenges of the integration and the potential effects in their medical practice.

This paper presents results of the overall analysis of semi-structured interviews conducted within general practitioners, nurses and specialist working at the diabetes care program. The aim was to determine common motivations and differences around inter-professional networks and identify mechanisms that explain how networks might affect patient outcomes and prescription behaviours at the integrated care context. Two research questions were addressed: From the health professionals' perspective, identify why social networks are affecting patient outcomes and prescription behaviours, and identify factors that may explain this effect.

## Materials and Methods

###  Context of the research

Counties Manukau (CM) is part of the Auckland city in New Zealand. More than 350 thousand residents are covered by the health system through the universal access model in this region. Counties Manukau District Health Board (CMDHB) and the Middlemore hospital receive support from the Ministry of Health to provide healthcare services. CMDH contracts primary care services with Primary Health Organizations (PHO), and they reimburse health providers and doctors 24. In 90s, CM had one of the most disproportionate increases in health needs. The most relevant factors that explained this phenomenon were the high demand in chronic care such as diabetes and it's complications which is explained by the presence of socio economic disparities, and additionally a poor coordination between primary and secondary care interface 25. Recent studies shown that 8% of this population has diabetes. The prevalence is focused on Pacific Islanders (15%), Maori (10%), Asians (8%) and Europeans (5%) within other etnic groups. Counties Manukau has 14% of the patients with diabetes in New Zealand, the reason why that geographical area represent one of the national priorities for health improvement 26.

### Study participants

Health professionals involved in diabetes care within primary and secondary care interface in CMDHB were invited. Respondents were general practitioners and practices nurses in primary care, and diabetes nurses specialist and endocrinologist at the secondary care (Middlemore hospital)(Table 1).

**Table 1.**
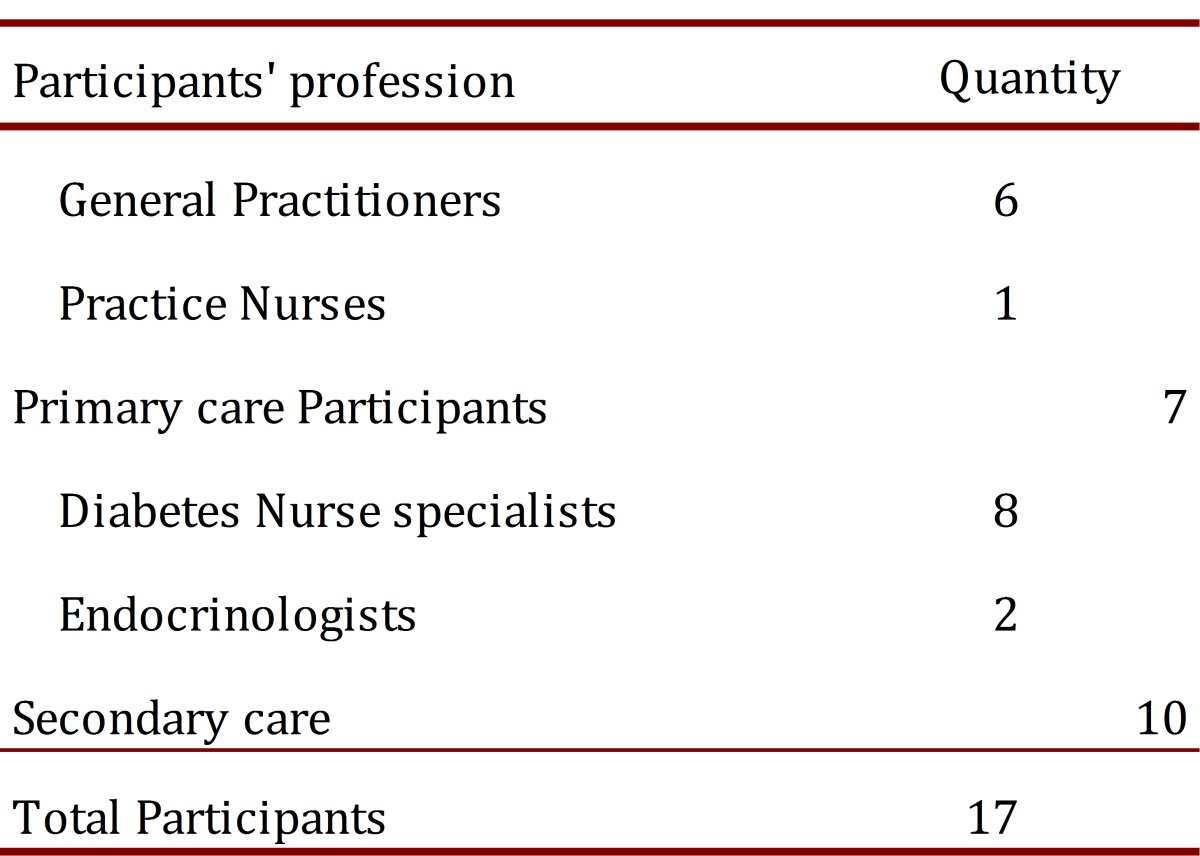
Participants in the qualitative analysis. Distribution by profession and sector

### Study design and data collection

This was a qualitative study. Semi-structural interviews were used for data collection. Two main questions were focused in the interview: Do you think social networks might affect patient outcomes and prescription behaviours in this particular context, if so, what are the mechanism that might explain this effect?. Interviews were performed prior consent form at the respondents room. Interviews, lasting 30-60 min, took place in the field of the health workers prior consent form. Each interview was transcribed verbatim independently in Microsoft^®^ Word version 2010 by an experienced technician who had signed a confidentiality agreement, according to the ethics approval. The written material from the transcribed interviews was the central element to reporting the findings analysis. Each transcription was anonymized.

### Data analysis

Using all interviews transcripts coding were performed in order to find the content in the units of analysis. The extraction of knowledge was based on a themes-clustering process. The foundation of this technique is the identification of the relationships between thematic networks. That means key themes; concepts and ideas were identified by data within the unit of analysis found in the literature review. Each unit of analysis was analyzed separately and identified themes and concepts that were visually presented as "concept maps" showing the main relationships between them. The method used simulates forces between the concepts. The map offered a graphical visualization that presents concept frequency, concept connectedness, direct interconcept and relative co-occurrence frequency, and total (direct and indirect) interconcept co-occurrence (proximity)(see www.leximancer.com.au for more detail) 27.

Each sphere represents a theme and within them are concepts. The name of each theme is in the color of the sphere while the name of the concept is in black. There are edges that show connections and paths between concepts. The greater the central position, the size and color of each sphere, the more important the theme is.

### Ethical approval

Ethical approvals were obtained as a part of the doctoral thesis for each stage of the study. Northern X Regional Ethics Committee ethical approval NTX/11/EXP/150 dated 19/07/2011 and Counties Manukau District Health Board research approval 1233 dated 16/02/2012.

## Results

Seventeen health workers (4 men and 13 women) participated in the semi-structured interviews from 28 extended invitations. The average age was 53 ys with a range from 43 to 63 yrs. Seven participants worked in primary care and ten in secondary care.

**Figure 1.**
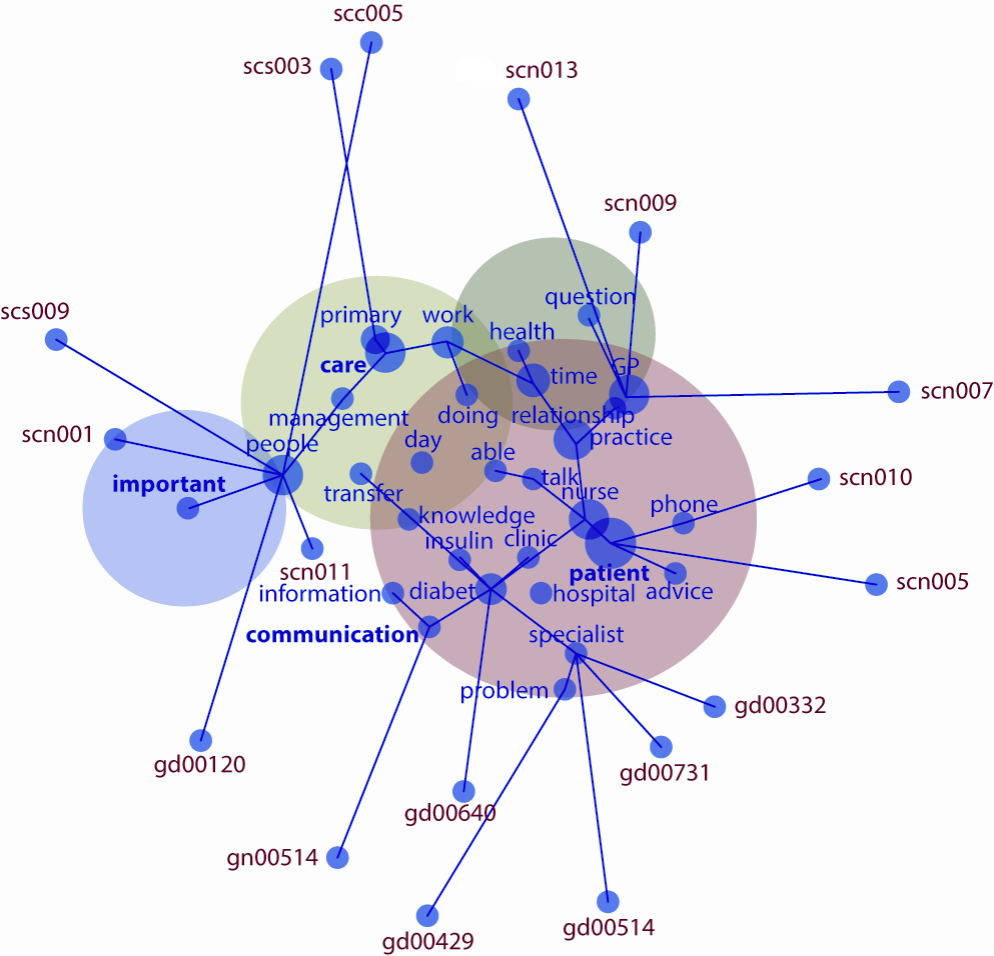
Conceptual map from general analysis: Distribution of semanthic groups by professions. Key themes are represented by circles; red circle are "patient", green circle are "care", dark green circle represent "communication" and violet circle "important".

Firstly, it is important to highlight that all of the participants agreed with the statement that participation in social networks has effects on patient outcomes at the primary and secondary care interface. Secondly, the mechanism that might explain the ways inter-professional networks affect patient outcomes and prescription behaviours emerge from the conceptual map (Fig. 1).

This map shows four key themes (conceptual groups semantically related) ranked according to the relevance or connectivity: "patient" had 100% of connectivity within other semantic groups, "care" (49%), "communication" (18%) and "important" (3%). Additionally, it was identified how each respondent was closely related with each semantic group. The "gd" codes represent each general practitioner, "gn" practices nurses, "scn" diabetes nurse specialist and "scc" endocrinologists.

Primary care health professionals were closely related with "communication" and "important" semantic groups, while health professionals at secondary care were closely related with "patient" and "care", and two health professionals where closer to "care". These two professionals had in common management roles as an addition of their clinical duties.

## Discussion

There are two key findings in this study: the four semantic groups and the way health professionals are clustered into these groups. The concept "patient" emerged as a result of two elements: firstly, from the idea that better "relationships" between health professional affect patient outcomes and prescription behaviours in positive way. Secondly, based on the principle that patient is the focus of integrated care.

"Communication" was the second semantic group. The majority of the general practitioners were clustered into this group. That was relevant because they recognized the importance of the knowledge transfer from secondary care. "Insulin" concept is inside this group and linked with "problems" concept. This demonstrates the difficulties around insulin therapy in primary care, and the lack of confidence to manage insulin protocols as a result of the potential complications (hypoglycemia). "Communication" could be one of the ways to overcome this issue.

The concept "nurse" is located in the intersection between "patient" and "communication". "Nurse" emerged as a concept in recognition of their clinical role between primary care "practice" and secondary care "clinic". In this particular context diabetes specialist nurses have the ability to diagnose and treat patients with diabetes, they are mainly located in secondary care but they are strongly connected with primary care. Those nurses are confident in managing insulin therapy, one of the reasons why general practitioners recognized nurses as a valuable source of knowledge and "advise". The conceptual map also shown "communication" barriers between primary and secondary care: Diabetes specialist nurses suggested difficulties to contact general practitioners and discuss with them patient's cases. Primary care doctors were not "able" to answer in timely manner "phone" queries from diabetes specialist nurses.

"Care" concept also highlighted the relevance of the diabetes specialist nurses in this network. Key treatment actions are managed by this group of nurses, and they are key actors in diabetes management. That flourish in the concept "day" nurses and endocrinologist interact at secondary care in a daily bases about diabetes management. While health professionals at the primary care interact with less frequency. That means that there are differences in the frequency of the interaction within and across the interface.

"People" concept is in between "care" and "important" concepts. That point was highlighted by one of the endocrinologist and one diabetes specialist nurse. They considered that communication, collaboration and coordination between primary and secondary care are the main factors (important) that might explain the effects of the inter-professional networks in patient outcomes and prescription behaviours. Collaboration and coordination flourished as the most important factors, which enhance integration within levels of care. They consider that in order to improve these factors better communication is required within the network. And the ultimate goal is to smooth the progress of knowledge transfer that facilitates changes in prescription behaviour in primary care.

The above analysis supports that general practitioners and nurses practices are seekers of information in secondary care (on how to treat patients with diabetes), while secondary care health professionals act as advisers and knowledge providers. That supports the statement that the direction of the influence in this network comes from secondary care to primary care. Additionally, current study highlights the "bridge" role of the diabetes nurse specialists. They are connecting primary and secondary care using their particular clinical skills, advising and supporting general practitioners in making decision process. Also the study suggests that there is need to improve the channels for communication in order to facilitate knowledge transfer in both ways. It is also highlighted that collaboration, coordination and communication are the main mechanisms that might explain how inter-professional networks affect patient outcomes and prescription behaviours.

## Conclusion

Through semantic analysis and conceptual mapping, it was possible to depict how inter-professional networks might affect patient outcomes and prescription behaviours at the primary and secondary care interface. Key factors were found, but an additional semantic analysis inside each factor is needed in order to have a better understanding. This study presents general guidelines to consider integration within health professionals as a way to increase communication, knowledge transfer and positive effects in prescription behaviours and patient outcomes. However, this study highlights some challenges in implementing communication strategies. Integrated care is more than a strategy to connect various components in the health system; faces the challenge of breaking barriers to increase communication and collaboration between human beings.

This qualitative study was made on the whole information received. In order to analyze in more detail the points of the research questions, it is necessary to resort to qualitative analysis of fragments specific information. The methodological design and the way the sample was selected prevent the generalization of the findings. However, the transfer to similar contexts can be used at the discretion of the applicant.
